# Advancing Medical Training with Mixed Reality and Haptic Feedback Simulator for Acupuncture Needling

**DOI:** 10.3390/s25226934

**Published:** 2025-11-13

**Authors:** Kasunika Guruge, H. M. K. K. M. B. Herath, Nuwan Madusanka, Hi-Joon Park, Chang-Su Na, Myunggi Yi, Byeong-il Lee

**Affiliations:** 1 Industry 4.0 Convergence Bionics Engineering, Pukyong National University, Busan 48513, Republic of Korea; kasunikag@pukyong.ac.kr (K.G.); kasunkh@pukyong.ac.kr (H.M.K.K.M.B.H.); 2 Digital Healthcare Research Center, Pukyong National University, Busan 48513, Republic of Korea; nuwanv@pknu.ac.kr; 3 College of Korean Medicine, Kyung Hee University, Seoul 02453, Republic of Korea; acufind@khu.ac.kr; 4 College of Korean Medicine, Dongshin University, Jeonnam 58245, Republic of Korea; csna@dsu.ac.kr; 5 Division of Smart Healthcare, College of Information Technology and Convergence, Pukyong National University, Busan 48513, Republic of Korea

**Keywords:** haptic feedback, healthcare education, HoloLens 2, medical simulation, metahuman, mixed reality

## Abstract

Traditional acupuncture training often lacks consistent, objective feedback, while current extended reality (XR) solutions rarely include quantitative assessment. This study developed and evaluated a feedback-enabled mixed reality (MR) acupuncture simulator to improve skill acquisition through depth-responsive guidance. The system, used on Microsoft HoloLens 2, combines a MetaHuman-based virtual patient with expert-designed acupoint geometries. It provides depth-dependent vibrotactile cues via a wearable haptic device and calculates a composite score from normalized metrics, including insertion depth, angular deviation, tip-to-center distance, and task duration. Ten participants (eight novices and two experts) performed needle tasks at LI4, LI11, and TE3 across two sessions. Mean depth error decreased from 6.41 mm to 3.58 mm, and task time from 9.29 s to 6.83 s. At LI11, beginners improved in achieved depth (16.24 ± 1.88 mm to 19.74 ± 1.23 mm), reduced angular deviation (27.83° to 15.34°), and shortened completion time (38.77 s to 13.28 s). Experts outperformed novices (69.25 ± 21.64 vs. 56.26 ± 23.37), confirming construct validity. Usability evaluation showed a mean overall score of 4.46 ± 0.51 and excellent reliability (McDonald’s ω = 0.93). These results demonstrate that expert-informed scoring and depth-responsive haptic feedback substantially enhance accuracy, efficiency, and learning confidence, validating the system’s technical robustness and educational readiness for clinical acupuncture training.

## 1. Introduction

Acupuncture, a key therapeutic modality in Traditional Korean Medicine (TKM), has demonstrated clinical efficacy in managing pain and a range of systemic disorders. However, mastering acupuncture requires precise localization of meridians and acupoints, controlled insertion depth, and accurate needle manipulation [1,2]. Even minor technical deviations can lead to complications, such as tissue damage or organ injury, underscoring the importance of safe and structured training environments. Conventional teaching methods, such as lectures, 2D anatomical atlases, and peer demonstrations, offer a limited three-dimensional understanding and inconsistent feedback. Silicone or gel-based phantoms partially mitigate safety concerns but lack anatomical fidelity and haptic realism, while assessment remains largely observational and subjective [1,3]. These limitations underscore the need for immersive, measurable, and feedback-driven approaches to procedural training in acupuncture.

**Figure 1:**
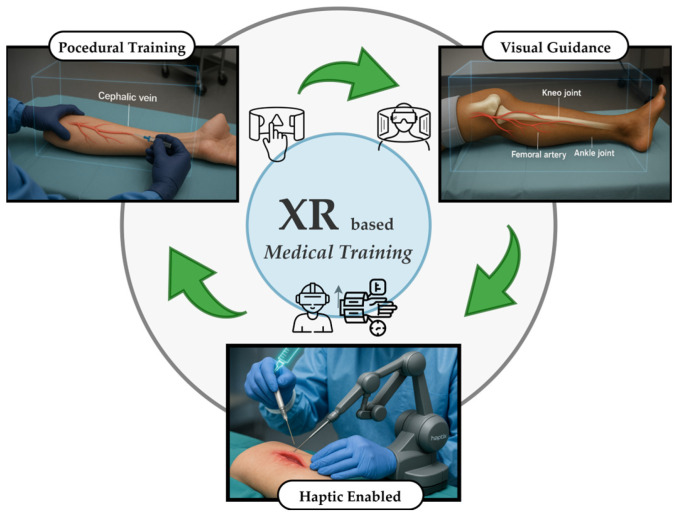
Overview of the existing XR approaches for medical training.

Recent advances in XR, encompassing augmented reality (AR), virtual reality (VR), and MR, have transformed medical education by enabling realistic and interactive simulations, as illustrated in Figure 1. In this study, MR refers explicitly to the interactive holographic environment implemented on Microsoft HoloLens 2, where virtual content is spatially anchored to the real world. The term XR is used more broadly to denote the collective domain encompassing VR, AR, and MR technologies.

Previous studies by Sung et al. [4], Kim et al. [5], Liu et al. [6], Jongbloed et al. [7], and Mitchell et al. [8] consistently demonstrated that XR enhances knowledge retention, procedural accuracy, and user confidence across various healthcare disciplines, including nursing, anesthesiology, and surgery. According to Han et al. [9], Fleet et al. [10], and Rubin et al. [11], XR facilitates deliberate practice, real-time feedback, and safe error correction, aligning with evidence-based learning principles. Among head-mounted displays, the Microsoft HoloLens 2 has been widely adopted for clinical simulation due to its spatial mapping capabilities, gesture tracking, and ability to integrate virtual content into real environments [12]. In surgical and anesthesia education, validated frameworks such as OSATS and GOALS have established the value of quantitative metrics, including task time, angular precision, and trajectory smoothness, in evaluating procedural competence [13,14,15,16,17]. These successes demonstrate the potential of XR for complex, precision-dependent procedures such as acupuncture.

XR-based training for needle-guided interventions has expanded rapidly. MR lumbar-puncture simulators using force feedback have produced measurable gains in depth control, confidence, and learning efficiency [18,19]. Similarly, VR-based lumbar puncture and spinal anesthesia systems that combine tactile cues with immersive visualization have improved procedural accuracy and user engagement [20,21,22,23,24,25]. MR-enhanced neurosurgical and neuraxial anesthesia trainers, which integrate holographic overlays and trajectory guidance, have been shown to improve spatial orientation and reduce task time in studies by Agathe et al. [21], Peng et al. [22], and Tanwani et al. [23]. Furthermore, in [18,19,20,21,22,23,24], the value of XR in reproducing realistic psychomotor demands was highlighted, particularly when paired with haptic feedback that reinforces depth awareness and tissue resistance. Despite such progress, most implementations focus on high-risk interventions (e.g., lumbar puncture, catheter placement) rather than on acupuncture, which demands subtler tactile perception and fine motor control.

By contrast, XR-based acupuncture systems remain in early development. Sun et al. [2] introduced an MR visualization platform that enables acupoint exploration via HoloLens; however, it lacks interactivity and assessment capabilities. Several investigations have examined educational and cognitive dimensions, for example, EEG-integrated VR environments for acupoint selection by Liao et al. [26], and AR-based mobile applications for anatomical recall by Zhang et al. [27], but these approaches provide limited procedural realism. Likewise, according to Chiou et al. [28], static AR acupoint tables and computer vision-based localization systems, as described by Yang et al. [29], enhance visualization but fail to facilitate hands-on training. Notably, nearly all prior acupuncture–XR systems omit haptic feedback, a component essential for realistic depth calibration and for conveying tissue penetration. Likewise, few incorporate quantitative, multi-parameter scoring, instead relying on post hoc instructor evaluation. In contrast, contemporary surgical simulators employ automated, objective scoring to deliver immediate, data-driven feedback [14,16,30]. This discrepancy highlights a critical gap between acupuncture education and other procedural domains.

Evidence from broader XR literature suggests that effective simulation platforms share three defining characteristics: (1) Task-specific, clinically grounded metrics, (2) Multidimensional performance assessment, and (3) Real-time, actionable feedback [14,16,31,32]. Translating these principles to acupuncture training requires a synchronized evaluation of insertion depth, angular alignment, lateral targeting, and temporal control elements that are rarely addressed in existing systems. Moreover, integrating tactile cues with immersive visual feedback could substantially enhance spatial memory and proprioceptive calibration during the insertion of needles.

Motivated by these gaps, this study proposes a MR-acupuncture training system that unifies expert-informed assessment with real-time haptic feedback to enhance procedural competence. Developed in Unreal Engine (UE) 5 and deployed on Microsoft HoloLens 2, the platform features a life-sized MetaHuman avatar, expert-derived acupoint models, and a wearable ESP32-based vibrotactile device providing depth-responsive vibration feedback. The system continuously captures key performance parameters, including needle insertion depth, angular deviation, tip-to-center distance, and task duration, and then synthesizes them into a composite, expert-weighted score. Unlike conventional models that rely solely on visual guidance, the proposed design enables multisensory feedback, reinforcing depth estimation through vibration intensity that is proportional to the depth of insertion.

The system incorporates two progressive training modes. The Beginner Mode displays visible acupoint markers to guide novices in spatial orientation, whereas the Advanced Mode conceals these cues, compelling reliance on anatomical recall and haptic sensation. Immediate visual and numerical feedback following each trial supports iterative self-correction, which aligns with the principles of deliberate practice pedagogy. The combination of visual immersion, tactile realism, and quantitative evaluation enables a closed-loop learning environment that mirrors the cognitive and sensory demands of authentic acupuncture procedures.

The novel contributions of this study are fourfold. First, it introduces a full-scale MR-compatible acupuncture simulator that integrates realistic virtual anatomy with natural hand interaction, providing lifelike procedural visualization that mirrors clinical needling contexts. Second, it incorporates expert-validated acupoint geometries and tolerance parameters to establish a robust foundation for quantitative, reproducible performance evaluation. Third, it presents a wearable haptic feedback interface that enhances depth perception through continuous, real-time vibration, enabling users to gauge insertion depth and prevent over-penetration intuitively. Fourth, it integrates a multi-parameter, expert-weighted scoring algorithm to objectively assess needling precision across spatial, angular, and temporal dimensions. Collectively, these features advance beyond prior XR-based acupuncture prototypes by combining high anatomical realism, quantitative feedback, and tactile guidance within a unified MR training framework.

By situating acupuncture within the broader framework of simulation-based medical education, this research advances the pedagogical shift toward quantitative, feedback-rich, and multisensory learning environments. The proposed system addresses not only the absence of tactile realism and objective scoring in previous XR acupuncture applications but also demonstrates the feasibility of combining expert knowledge, real-time analytics, and embodied interaction within a single, integrated framework. Consequently, it offers a promising foundation for standardized, scalable acupuncture training that aligns with contemporary trends in immersive healthcare education and competency-based assessment.

## 2. Methodology

**Figure 2:**
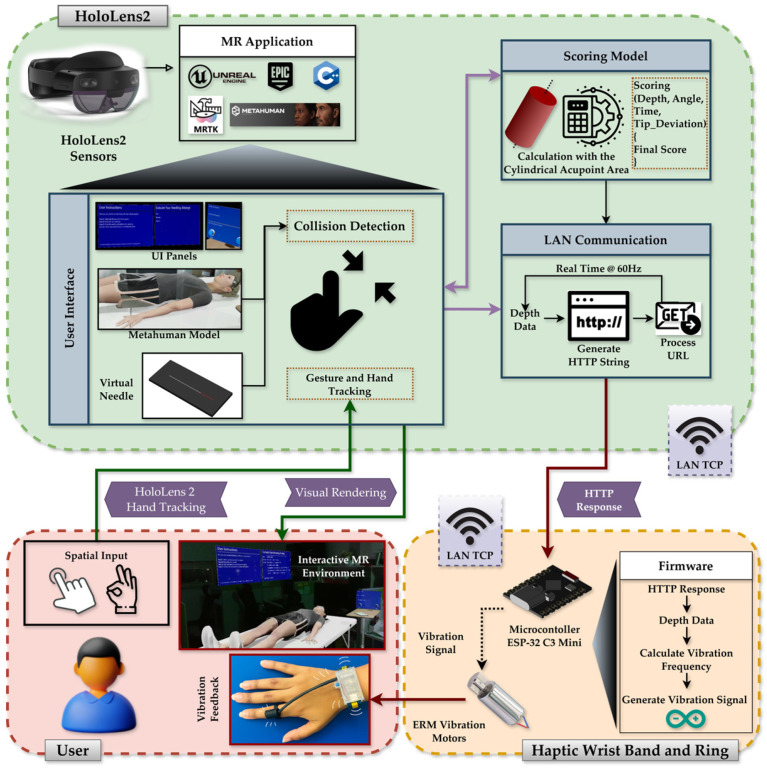
System architecture diagram showing the HoloLens 2 and haptic device hardware pipeline, MR environment, MRTK integration, MetaHuman and interaction subsystems, and real-time communication with the wearable haptic device for depth-based vibration feedback.

This section outlines the system architecture, hardware–software integration, acupoint modeling strategy, data capture mechanism, expert-informed scoring algorithm, and user evaluation mechanism of the system. The entire application was developed in UE 5.0 and deployed on the Microsoft HoloLens 2 platform. Core components include a high-fidelity virtual patient model (MetaHuman), a real-scale virtual needle, anatomically grounded acupoint markers, a custom-designed wearable haptic feedback device, and a modular performance evaluation pipeline, as illustrated in Figure 2.

### 2.1. Mixed Reality Application Design and Development

The system architecture comprises a MetaHuman-based virtual patient, a real-scale interactive needle, expert-defined acupoint models, and a wearable ESP-32 C3-driven vibrotactile device connected via TCP at a rate of 60 Hz. Together, these components create a synchronized visual-haptic training environment for procedural skill acquisition.

**Figure 3:**
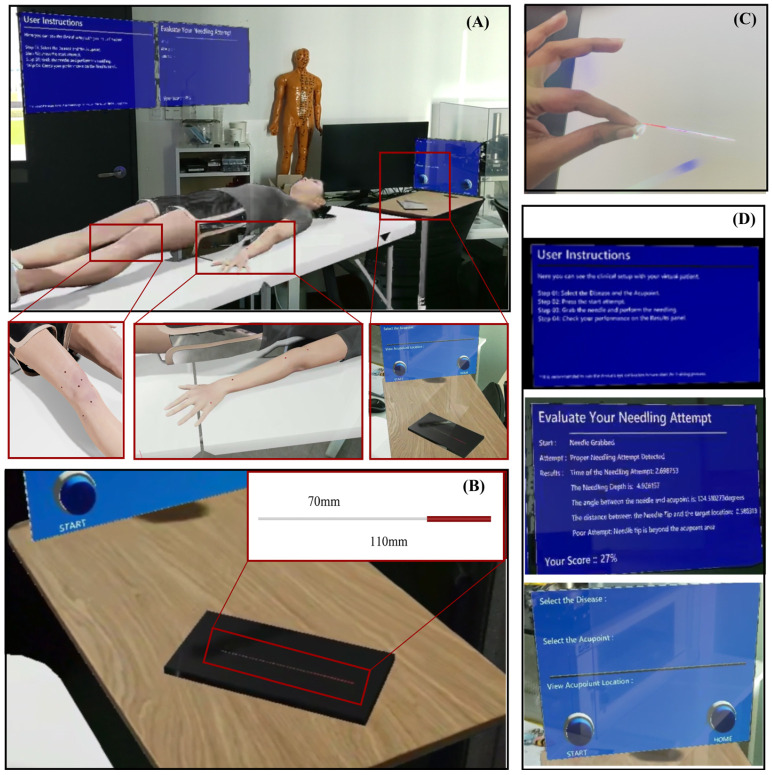
The MR acupuncture training environment and interaction components. (**A**) Complete virtual clinical setup spatially anchored to the real world, (**B**) Virtual acupuncture needle positioned on the bedside table for natural reach and grasp, (**C**) Bare-hand interaction view showing how users see and manipulate the virtual needle, and (**D**) Floating user interface panels.

The HoloLens 2 offers inside-out spatial mapping and articulated hand tracking, allowing for natural, bare-hand interaction without the need for external controllers. The virtual clinical scene, consisting of a bed and side table, is spatially anchored to the real environment, allowing the MetaHuman avatar to appear at life size and preserving realistic ergonomics for the learner, as shown in Figure 3A.

The virtual needle (see Figure 3B) was modeled as a slender 110 mm × 1 mm cylinder, approximating the dimensions of a clinical instrument. Gesture recognition via MRTK enables grasping, insertion, and release actions with the bare hand. As shown in Figure 3C, Users can freely approach and manipulate the virtual needle using hand gestures, replicating the posture of an authentic operator. To support usability, graphical interface panels (see Figure 3D) provide (1) Step-by-step task instructions, (2) Acupoint selection and session controls, and (3) Real-time performance feedback showing measured parameters and overall scores. These floating UI elements maintain visibility without obstructing the operative field.

**Figure 4:**
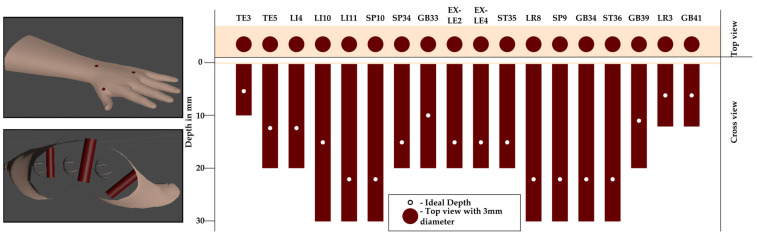
Cylindrical acupoint model representation. The top surface of the cylinder (top view) is aligned with the MetaHuman skin, while the remainder of the cylinder extends internally. The visible circle serves as surface guidance, and the central axis defines the ideal trajectory and optimal insertion depth.

Acupoint modeling was derived from structured questionnaires completed by three licensed practitioners, defining safe depth ranges, insertion angles, and tolerance margins for 18 clinically relevant acupoints. Expert-defined parameters, summarized in Table 1, establish clinically realistic bounds for evaluation. The marker’s central axis denotes the ideal insertion path, and deviations beyond the defined tolerances are automatically penalized during scoring. Each acupoint was represented as a cylindrical marker embedded beneath the MetaHuman skin surface (see Figure 4). The visible top view of the cylinder corresponds to the traditional surface marking, while the internal cylinder encodes permissible insertion depth and angular trajectory.

**Figure 5:**
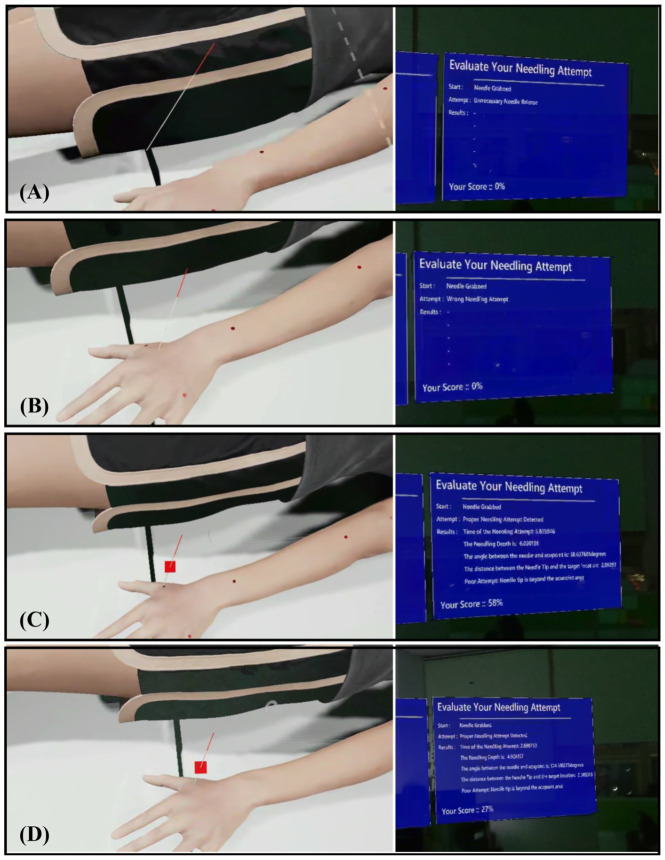
System classification of needle insertion outcomes and training mode visuals. (**A**) Unnecessary release, (**B**) Incorrect insertion, (**C**) Valid insertion, and (**D**) Advanced Mode visualization, which features hidden acupoint markers.

A collision-based detection model in UE governs system interaction. When the user releases the virtual needle, the system classifies the event as (1) Unnecessary release (see Figure 5A), (2) Incorrect insertion, such as skin contact without acupoint intersection (see Figure 5B), or (3) Valid attempt intersecting an acupoint marker (see Figure 5C). According to Algorithm 1, only valid attempts trigger quantitative evaluation. For these cases, four parameters were automatically recorded: insertion depth, angular deviation, tip-to-center distance, and insertion time. These data served as inputs to the expert-informed scoring model as described in Section 2.3. The deliberate design choice to evaluate performance only upon release mirrors real-world practice, where assessment occurs after the needle is positioned.
**Algorithm 1** Needle Release Event Handling and Data Capture**Require:** Needle collider *C_N_*, release event *R* **Ensure:**   Attempt classification *C*, performance metrics {*D*, *A*, *T*, *IT*} 1: *b*_1_ ← CollideSurface (CN) ▷ collision with any surface? 2: **if** ¬ *b*_1_ **then** 3:  *C* ← “Unnecessary Release” 4:  **return** (*C*, *∅*) 5: *b*_2_ ← CollideSkin (CN) ▷ collision with skin? 6: *b*_3_ ← CollideAcupoint (CN) ▷ intersects acupoint marker? 7: **if** *b*_2_ ∧ ¬ *b*_3_ then 8:  *C* ← “Wrong Needling Attempt” 9:  **return** (*C*, *∅*) 10: **if** *b*_3_ **then** 11:  *C* ← “Proper Needling Attempt” 12:  *D* ← ComputeDepth (*S_skin_*, *p_tip_*) 13:  *A* ← ComputeAngle ($\vec{a}$_needle_, $\vec{a}$_ideal_) 14:  *T* ← ComputeOffset (*p_tip_*, *p_ap_*(*D*)) 15:  *IT*← ComputeDuration (*t_grab_*, *t_release_*) 16:  RecordMetrics (*D*, *A*, *T*, *IT*) 17:  TriggerScoring (*D*, *A*, *T*, *IT*) 18:  **return** (*C*, {*D*, *A*, *T*, *IT*})

To support adaptive learning, the platform incorporates two training modes. In (1) Beginner Mode, Acupoint markers were visibly rendered on the MetaHuman surface to guide spatial localization and provide immediate visual feedback (see Figure 5C). In (2) Advanced Mode, markers were hidden (see Figure 5D), requiring reliance on anatomical memory and haptic cues from the wearable device to judge insertion depth. Regardless of mode, the system displays post-trial metrics and a composite performance score, enabling self-correction through iterative practice.

The wearable haptic device (discussed in Section 2.2) operates in tandem with the virtual system to provide depth-contingent vibration feedback during needle insertion. Accordingly, the system continuously calculates a real-time penetration depth ($d_{hap}$) whenever the virtual needle intersects the MetaHuman skin surface. This depth value represents the instantaneous tip-to-skin distance along the acupoint’s insertion axis and is used solely for generating haptic feedback, independent of the performance-scoring framework. When the needle is not overlapping with the MetaHuman or not in the grabbed state, $d_{hap}$ is set to 0, ensuring that no vibration occurs during idle periods. The value is updated in real time at the HoloLens frame rate of 60 Hz. For each acupoint, the expert-defined maximum safe depth ($D_{max}$) from Table 1 serves as a safety reference. The current $d_{hap}$ value and its corresponding $D_{max}$ are combined into an HTTP string and transmitted to the ESP32-C3 microcontroller via TCP communication, with the microcontroller acting as a server that continuously listens for client updates. The VaREST plugin in Unreal Engine processes the URL request using the GET method. Upon receiving these values, the haptic module produces light vibration pulses for shallow insertion, progressively stronger pulses for deeper penetration, and a distinct buzz if $d_{hap}$ exceeds $D_{max}$. In contrast, the final insertion depth recorded at the needle-release event is separately used in the quantitative scoring model described in Section 2.3. This clear separation between real-time tactile feedback and post-trial scoring ensures consistent sensory guidance without interfering with objective evaluation.

Overall, the system establishes a closed-loop, sensor-based training framework in which visual, tactile, and quantitative feedback operate synergistically. By grounding the virtual geometry in expert-validated anatomical data and coupling it with real-time depth feedback, the design ensures that users can practice accurate needle placement under clinically meaningful constraints. The resultant platform supports consistent, measurable, and immersive acupuncture training aligned with contemporary simulation-based education principles.

### 2.2. Haptic Feedback System Development and Integration

In conventional MR interactions, users rely solely on visual feedback, as virtual objects provide no physical resistance or tactile sensation. During acupuncture simulation, the absence of haptic feedback makes it difficult to estimate insertion depth or perceive contact with the skin, especially when manipulating virtual instruments, such as needles. To address this limitation, a haptic feedback system was incorporated to recreate the sense of touch that was missing. When the virtual needle tip collides with the MetaHuman’s skin, the wearable device delivers a vibration pulse corresponding to the contact event, allowing users to sense the interaction physically. As the needle penetrates deeper, the vibration intensity increases progressively, providing depth-aware tactile cues that help users align their visual perception with the expected physical sensation of insertion.

**Figure 6:**
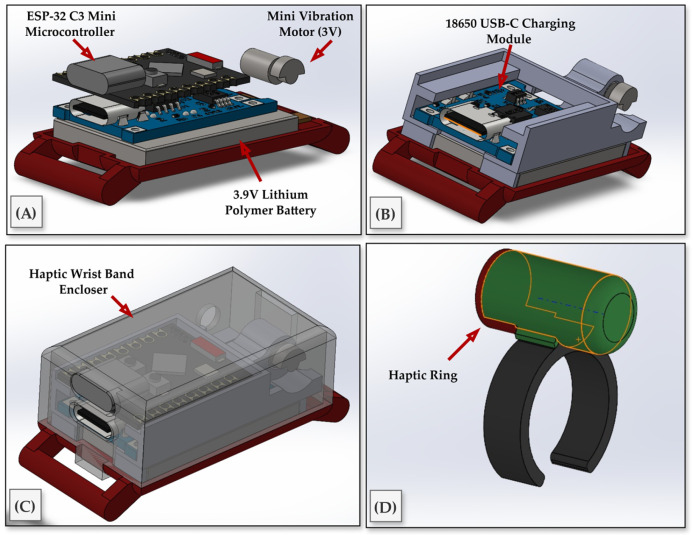
Hardware design and integration of the wearable haptic feedback device. (**A**) Internal components include the ESP32-C3 Mini microcontroller, a 3.9 V Lithium-polymer battery, and a 3 V micro ERM vibration motor. (**B**) USB-C charging module integrated into the compact PCB frame. (**C**) Fully assembled wrist-worn enclosure with transparent outer casing. (**D**) Haptic ring housing the vibration motor, worn on the index finger and connected to the wristband via a thin cable.

The methodology for the virtual-needling haptic device centered on a compact, wearable implementation available in two interchangeable form factors, a finger ring and a wrist-band module, driven by an ESP32-C3 Super Mini microcontroller and a 3 V eccentric-rotating-mass (ERM) vibration motor as the tactile actuator. To achieve this, we designed a self-contained electronics stack that preserves access to the USB-C port and header pins, while arranging the controller, charging board, battery, and motor in a low-profile enclosure (see Figure 6A–C). The actuator is housed in a cylindrical sleeve and secured to the fingertip with an open-C, size-adjustable ring that maintains skin contact for efficient vibrotactile coupling during motion (see Figure 6D). The housing was designed in CAD to position the electronics, battery, and motor, and to provide reliable cable relief and motor seating. These components were fabricated using a FormLabs SLA printer. To balance stiffness, precision, and translucency, the base and circuit holder were printed in FormLabs Black resin, chosen for its dimensional stability, which is suitable for snap-fits and threaded inserts. Meanwhile, the protective enclosure was printed in Clear resin, allowing for visual inspection of the electronics and battery without disassembly. The ring itself was also printed in Black resin to maximize durability under bending and repeated donning.

The electronics were selected to minimize footprint and simplify charging. A single-cell 3.7 V lithium-ion polymer battery powered a TP4057-based USB-C charging and protection module; the module’s OUT terminals provided a protected rail for the ESP32-C3 Super Mini and the motor driver stage. The ESP32-C3 was chosen for its integrated Wi-Fi and compact form factor, and it was used to synthesize the vibration drive signals from HTTP responses generated by the HoloLens.

During operation, a virtual simulation computed the instantaneous needle penetration depth (*d*) and the acupoint-specific maximum depth (*D_max_*). These quantities were transmitted from the virtual system to the wearable device; both systems were associated with the same access point to minimize routing overhead and reduce latency. Upon receipt of the HTTP payload, bounds were validated, and depth was mapped to a target vibration frequency via a linear law (Equation (1)):

(1) $$f\left(d\right)=\left(\frac{f_{max}}{D_{max}}\right)\cdot d,\;where\;0\leq d\leq D_{max}$$

In practice, the vibrotactile motor operated within an effective frequency range of 5–200 Hz, corresponding to low frequency for shallow insertion and progressively stronger vibration at greater depths. These parameters were determined empirically to ensure perceptible yet comfortable stimulation across all users while maintaining low power consumption. This scaling ensured that zero penetration yields no vibration, whereas full-scale penetration at a given acupoint produced a consistent maximum frequency *f_max_*, independent of the absolute value of *D_max_*.

As shown in the logic pipelines of Algorithm 2, the firmware employed a rate-limited controller that filters noisy depth updates and adjusts the vibration frequency to prevent perceptible stepping. A moderate update cadence preserved natural haptic continuity while avoiding excessive network traffic. On the hardware side, the ESP32-C3 executed a command to drive an ERM motor. For the ring configuration, the motor was housed in a cylindrical cradle to maximize mechanical coupling to the finger pad.
**Algorithm 2** Vibration Pulse Generation**Require:** constants *D_max_*, *f_max_* **Ensure:**   vibration at f = (f_max_/D_max_) · d when enabled; otherwise LOW  1:   *d* ← 0; *f* ← 0; *enabled* ← **false**  2:   *outputHigh* ← **false**; Δ*t_half_* ← 0; *t_next_* ← 0  3:   **procedure** Recalc  4:  *f* ← max(0, (*f_max_*/*D_max_*) · *d*)  5:  if ¬ enabled ∨ *f* = 0 **then**
  6:    Δ*t_half_* ← 0; *outputHigh* ← **false**; DigitalWriteLow()  7:    **return**  8:  Δ*t_half_* ← 1/(*2f*); *t_next_* ← Now() + Δt_half_; **return**  9:   **procedure** Start  10:  *enabled* ← **true**; Recalc  11: **procedure** Stop  12:  *enabled* ← **false**; Recalc  13: **procedure** SetDisplacement(*d_new_*)  14:  *d* ← min(max(0, *d_new_*), *D_max_*); Recalc  15: **function** MainLoop  16:  HandleHTTPRequests()  17:  if *enabled* ∧ Δ*t_half_* > 0 ∧ Now() ≥ *t_next_* **then**  18:   *outputHigh* ← ¬ *outputHigh*  19:   if *outputHigh* **then**
  20:    DigitalWriteHigh()  21:   else   22:    DigitalWriteLow()  23:   *t_next_* ← *t_next_* + Δ*t_half_*  24:  **return**          ▷ loop MainLoop continuously

**Figure 7:**
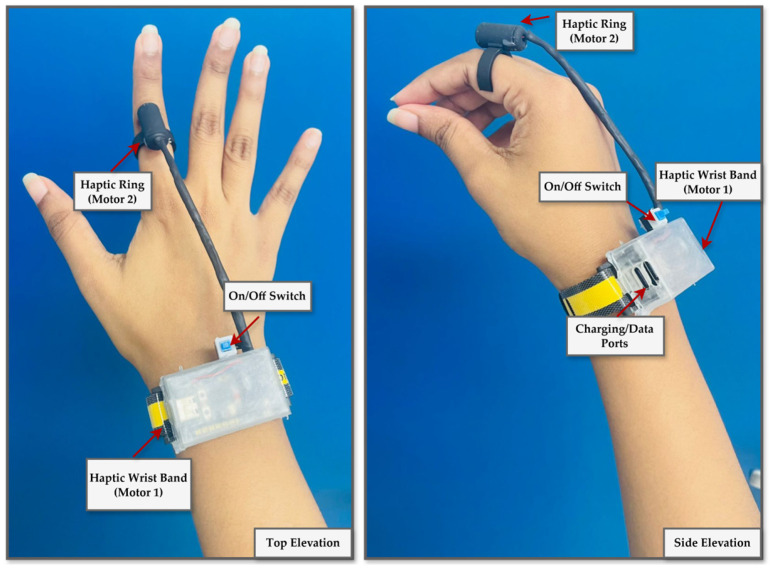
Final design of a wearable haptic feedback device. The ring configuration features a vibration motor on the fingertip for high tactile precision. At the same time, the wristband variant positions the motor over the radial or ulnar side to enhance comfort and stability during needle insertion tasks.

The final wearable designs comprise a finger-mounted vibration ring and a wireless wristband that enable unobstructed, freehand hologram interaction and the reliable delivery of depth-responsive tactile feedback, as illustrated in Figure 7.

### 2.3. Expert-Driven Scoring Model

The acupuncture training performance evaluation system was developed based on the expert-defined tolerances collected through the questionnaire study. For each acupoint, the experts specified a target insertion depth and an acceptable tolerance range, the ideal insertion angle with maximum allowable deviation, and the maximum lateral offset of the needle tip from the acupoint center. These values provided clinically grounded thresholds that guided the formulation of a multi-parameter scoring model (see Table 1).

The scoring framework comprises four normalized component scores, each scaled between 0 and 100, which together capture the multidimensional nature of acupuncture needling performance: depth accuracy, angular precision, spatial targeting, and temporal control.

1. Depth Score: Depth accuracy was modeled as
  (2) $$S_{d}=\left\{\begin{array}{c} \;\;\;\;\;\;\;\;\;\;\;\;\;100,\;\;\left|d_{u}-d_{target}\right|\;\leq \;d_{tol}\\ 100\cdot \left(1-\frac{\left|d_{u}-d_{target}\right|-d_{tol}}{d_{max}-d_{min}}\right),\;\;\;Within\;bounds\\ 0,\;\;otherwise \end{array}\right.$$
  where *d_u_* is the user’s insertion depth, *d_target_* is the ideal depth, *d_tol_* is the expert-defined tolerance, and *d_min_*, *d_max_* are the lower and upper bounds of safe insertion. Here, the expert-defined tolerance (*d_tol_* = ±2 mm) represents the clinically acceptable deviation from the target depth. The minimum (*d_min_*) and maximum (*d_max_*) depths define the absolute safe boundaries based on anatomical constraints. During scoring, depth errors within *±d_tol_* are rewarded with full points, whereas insertions beyond this range but within [*d_min_*, *d_max_*] incur proportional penalties. Insertions exceeding *d_max_* are automatically scored as 0 to reflect unsafe over-penetration.

2. Angle Score: Angular deviation from the ideal trajectory was calculated as
  (3) $$S_{\alpha }=\left\{\begin{array}{c} \;\;\;\;\;\;\;\;100,\;\;\;\theta \leq \;\theta_{tol}\\ 100\cdot \left(1-\frac{\theta -\theta_{tol}}{45-\theta_{tol}}\right),\;\;\;\theta \leq \;45{}^{\circ}\\ 0,\;\;\theta >45{}^{\circ} \end{array}\right.$$
  where *θ* is the deviation from the ideal angle, and *θ_tol_* is the expert-defined angular tolerance.

3. Weighted Accuracy: Since experts indicated that depth and angle should carry equal importance for every acupoint, a weighted accuracy score was computed as
  (4) $$A=\omega_{d}S_{d}+\omega_{\alpha }S_{\alpha }$$
  with default weights *ω_d_* = *ω_α_* = 0.5.

4. Tip Position Score: Spatial targeting was assessed based on the Euclidean distance of the needle tip from the acupoint center, compared to the permissible radius (*R* = 1.5 mm).

(5) $$P_{t}=\left\{\begin{array}{c} 1.00,\;\;r\;\leq R\\ 0.75,\;\;r\;\leq 2R\\ 0.50,\;r\;\leq 3R\\ 0.25,\;r>3R \end{array}\right.$$

5. Time Score: The temporal performance was scored against expert-recommended insertion times, with an optimal range of 10–20 s and a maximum acceptable limit of 60 s.

(6) $$S_{t}=\left\{\begin{array}{c} 1.00,\;\;t<20\;s\\ 0.75,\;\;20\;s\;\leq t<30\;s\\ 0.50,\;\;30\;s\;\leq t<40\;s\\ 0.25,\;\;40\leq t<60\;s\\ 0.0,\;\;t\;\geq 60\;s \end{array}\right.$$

6. Final Score: The overall performance score was computed as

(7) $$A_{final}=A\cdot \frac{\left(S_{t}+P_{t}\right)}{2}$$

**Figure 8:**
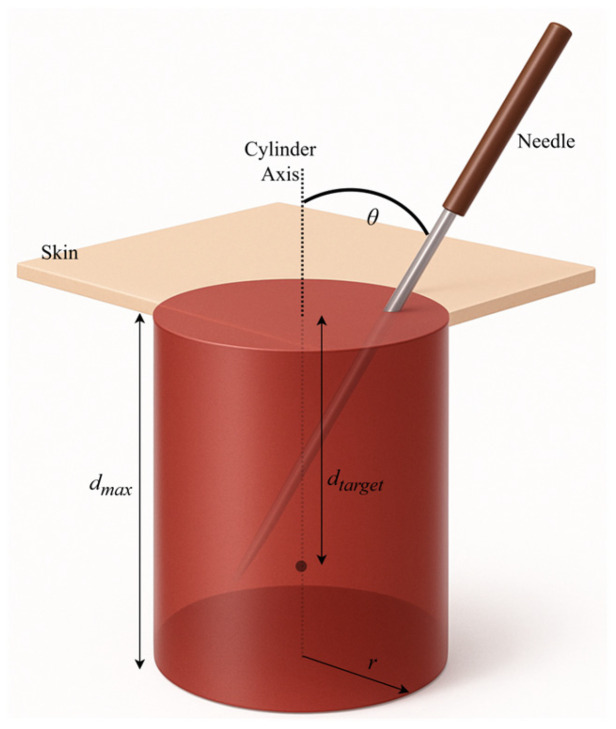
Visualization of the schematic of composite score calculation parameters.

This formulation integrates weighted accuracy (depth and angle) with temporal and spatial control, producing a balanced measure of needling competence. The complete penalty functions for depth, angle, tip location, and timing are visualized in Figure 8.

### 2.4. Usability Experiment Design

The primary objective of the user experiment was to demonstrate that the MR acupuncture needling trainer, integrated with a depth-responsive haptic device, is usable and capable of producing measurable learning effects within short, repeated sessions. This study focused on system-level feasibility, usability, and metric fidelity, rather than on evaluating clinical skill transfer to acupuncture trainees. Accordingly, the evaluation assessed user progress and interaction performance within the fully integrated MR–haptic environment, to validate the complete training system under realistic operating conditions.

#### 2.4.1. Participants

A total of ten participants were recruited (Table 2). Eight were beginners without prior experience in acupuncture needling, but they were familiar with the theoretical background through books or demonstration videos. Two were experts, defined as medical students with prior exposure to acupuncture practice in clinical or educational settings. All participants were well-experienced in using MR headsets and HoloLens 2, which allowed the study to focus on interaction with the needling trainer rather than acclimatization to immersive technology. Recruiting MR-familiar but non-clinical users was appropriate for this early-phase evaluation, as the aim was to establish usability and data reliability. This approach aligns with prior VR/MR healthcare-related studies, which first assess feasibility using convenience samples before moving to medical learners. This initial usability study was therefore performed with MR-familiar participants to isolate system-interaction factors; however, future validation will target medical trainees and clinical learners to evaluate educational transfer and curricular integration.

#### 2.4.2. Experimental Design and Tasks

The experimental protocol consisted of two sessions separated by 24 h. Each participant was provided 30–60 min of free practice to familiarize themselves with the virtual patient, acupoint markers, and haptic feedback. The haptic depth feedback was kept active in all sessions to maintain uniform experimental conditions and to evaluate user learning within the complete MR–haptic training environment rather than to isolate the independent effect of the haptic feedback module. In each session, participants performed ten consecutive needling attempts at three commonly used training acupoints: TE3, LI4, and LI11, which represent shallow, medium, and deep insertion depths, respectively. This produced 30 trials per session and 60 trials per participant across both sessions, yielding 20 repeated attempts per acupoint. An overview of the design, including participants, acupoints, and trial counts, is summarized in Table 3.

The system automatically logged each attempt. For valid insertions, the following metrics were recorded: insertion depth (mm), needle angle (°), tip-to-center distance (mm), insertion time (s), and the composite weighted scores derived from expert input. Errors, including needle drops and wrong insertions during a single trial, were also recorded. Additional measures included preparation time from the start of the session to the first needle grasp and total trial duration.

#### 2.4.3. Usability Questionnaire

Upon completion of both training modes, all participants were administered a structured usability questionnaire designed to evaluate multiple dimensions of user experience with the MR acupuncture training system. The instrument was an adapted version of the USE questionnaire, comprising 15 items rated on a 5-point Likert scale (1 = Strongly Disagree, 5 = Strongly Agree). Items were grouped a priori into six theoretical factors: Usefulness (UF), Ease of Use (EOU), Ease of Learning (EOL), Satisfaction (SAT), Interaction Naturalism (INT), and Haptic Feedback (HAP). Each factor was represented by two to three items reflecting distinct but related constructs of system usability and interaction quality. In addition to the quantitative items, the questionnaire included open-ended questions to elicit qualitative feedback regarding perceived realism of the needling task, comfort of the haptic device, and suggestions for system improvement. This mixed-methods approach was intended to capture both measurable usability outcomes and nuanced user perceptions, particularly in relation to novel features such as wearable haptic feedback.

## 3. Results

**Figure 9:**
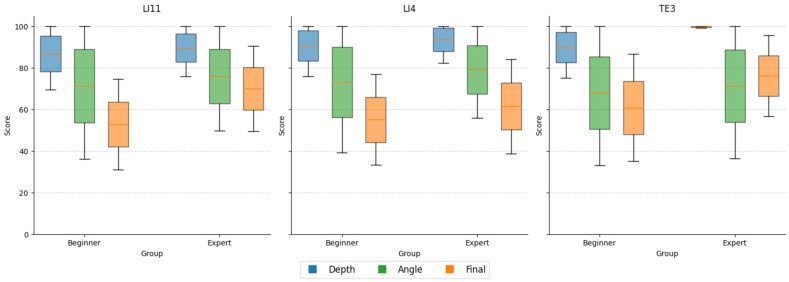
Comparison of performance metrics between beginners and experts. Boxplots illustrate differences in depth score, angle score, and composite final score across all trials for beginner (n = 8) and expert (n = 2) groups. Experts achieved higher final scores and exhibited reduced variability in depth accuracy, whereas angular deviation was the most distinguishing factor in group performance.

A total of 600 valid trials were collected across all participants, comprising 480 beginner and 120 expert trials. The expert-weighted scoring model demonstrated robust construct validity, as experts consistently outperformed beginners in composite performance, angular precision, and consistency of depth control on all three acupoints (see Figure 9). While both groups achieved similarly high mean depth scores (experts: 94.34 ± 10.94; beginners: 89.09 ± 15.58), experts displayed notably lower variance, indicating more controlled and repeatable insertions. The angle score contributed most strongly to group differentiation, with experts attaining a mean of 75.49 ± 28.45 compared to 70.86 ± 34.65 for beginners. Notably, the composite final score was significantly higher among experts (69.25 ± 21.64) than beginners (56.26 ± 23.37), confirming the scoring model’s ability to detect experience-based performance distinctions (Table 4).

As shown in Table 5, the procedural parameters, including insertion depth, angular deviation, and task duration, showed consistent improvement across the 20 trials for each acupoint, indicating clear learning progression among beginners.

For LI11, mean insertion depth increased from 16.24 ± 1.88 mm in early trials (1–5) to 19.74 ± 1.23 mm in late trials (16–20), approaching the expert-defined target of 21.25 ± 2 mm. Although the mean insertion depth for LI11 (19.74 mm) appears slightly below the nominal target of 21.25 mm, it remains within the expert-defined acceptable range when considering the 2 mm tolerance used for performance evaluation. This outcome suggests that participants can approximate the clinically relevant depth despite having limited prior needling experience. The marginally shallower insertions may reflect cautious behavior, particularly for LI11, which requires deeper penetration than the other tested points, and are consistent with a natural tendency among novices to avoid over-insertion. As this study involved MR-familiar but acupuncture-naïve participants, the observed result supports the system’s capacity to facilitate depth control learning rather than representing a task failure. Simultaneously, angular deviation improved markedly, from 27.83 ± 16.62° to 15.34 ± 12.66°, converging toward the permissible ±15° range. Duration also declined sharply, from 38.77 ± 14.68 s to 13.28 ± 5.53 s, well within the 20-s expert threshold.

Similar trends were observed for LI4 and TE3. At LI4, the mean depth increased from 8.30 ± 1.90 mm to 11.72 ± 1.29 mm, approaching the clinical target of 12.5 ± 2 mm, while angle deviation dropped from 27.79 ± 15.73° to 14.84 ± 10.03°, and task time improved from 42.90 ± 18.17 s to 12.05 ± 4.67 s. At TE3, where the target depth was shallower (5.5 ± 2 mm), early trials showed systematic overshooting (8.25 ± 1.77 mm), which was corrected to 5.63 ± 1.63 mm by the end of the sequence. Angular deviation and time followed similar improvements, reducing from 28.07 ± 14.20° to 15.91 ± 12.01° and from 46.64 ± 19.47 s to 13.03 ± 5.06 s, respectively (see Table 5). These progressive refinements demonstrate the system’s efficacy in promoting short-term skill acquisition aligned with expert performance boundaries.

**Figure 10:**
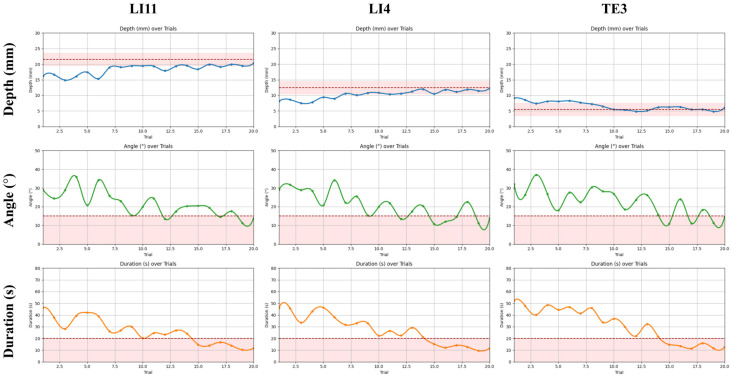
Learning curves of procedural performance over 20 trials in beginners with the shaded tolerance band and dashed safety limits. Trends for insertion depth, needle angle, and task duration are plotted across 20 consecutive trials at three acupoints (LI11, LI4, TE3). Shaded bands represent expert-defined acceptable thresholds for each parameter (depth: LI11 = 21.25 ± 2 mm, LI4 = 12.5 ± 2 mm, TE3 = 5.5 ± 2 mm; angle: 0–15°; duration ≤ 20 s).

The learning curves in Figure 10 further illustrate this convergence, showing consistent reductions in depth error, angular deviation, and task duration across trials. Notably, all three metrics stabilized within the expert-defined acceptable ranges by approximately the 15th trial for most participants. The shaded threshold bands provided explicit training targets. The participants’ trajectories clearly trended toward and plateaued within these bands, indicating reliable internalization of procedural norms and stabilization of motor performance through the MR training system.

Concomitantly, error rates and interface handling efficiency improved across sessions, further confirming enhanced procedural fluency. As summarized in Table 6, the average number of unnecessary needle droppings per five-trial block declined from 2.54 to 0.62, and anatomically incorrect insertions dropped from 3.44 to 1.08. Mean preparation time, defined as the interval from task initiation to first needle grasp, was reduced from 10.47 s to 3.11 s. These reductions indicate not only increased task familiarity but also a decrease in cognitive and operational load, which are essential for transitioning from early exploration to confident and efficient procedural execution.

**Figure 11:**
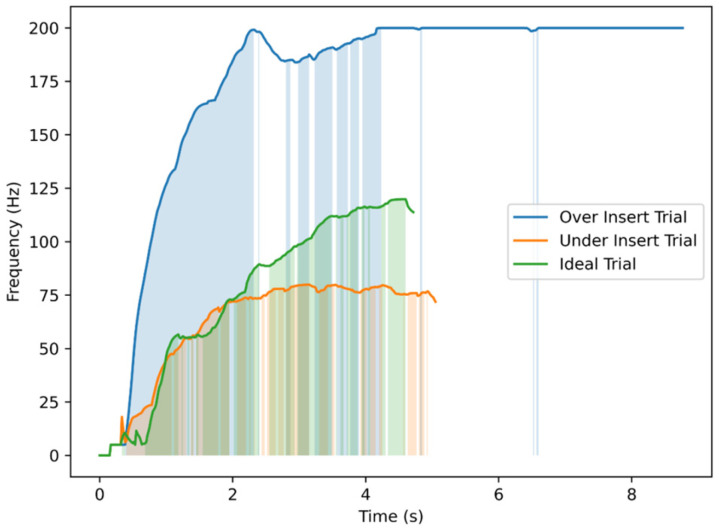
Vibrator motor command frequency over time for three representative TE3 insertion trials. Each trial corresponds to a different outcome in terms of needling depth: an overshoot exceeding the maximum safe depth, an ideal insertion reaching the expert-defined target depth, and an undershoot remaining below the minimum acceptable depth. Shaded regions indicate time intervals where vibration frequency increased in response to needle progression.

While the scoring data captured accuracy and learning progression, the influence of real-time tactile cues provided by the haptic device on depth perception was further investigated. The analysis of vibration frequency patterns during trials provided insights into how haptic feedback assisted users in estimating needle depth. Figure 11 illustrates three representative cases of the trials performed on TE3: an under-insertion (shallow), an ideal insertion, and an over-insertion trial. The vibration frequency increased progressively as the needle advanced, but the relationship was not linear, reflecting the natural variability and discontinuity of hand motion during insertion. In the over-insertion trial, the continuous high-frequency vibration at the maximum depth served as a corrective cue, helping users recognize and limit excessive penetration. In contrast, the ideal trial exhibited a gradual and well-regulated frequency rise that stabilized near the target depth, indicating practical depth estimation through tactile cues. The shallow trial remained at lower frequencies, corresponding to insufficient penetration and limited haptic stimulation. Overall, these results suggest that depth-dependent vibration feedback not only conveys proximity to the target but also supports intuitive self-correction, thereby improving control over insertion depth during training.

**Figure 12:**
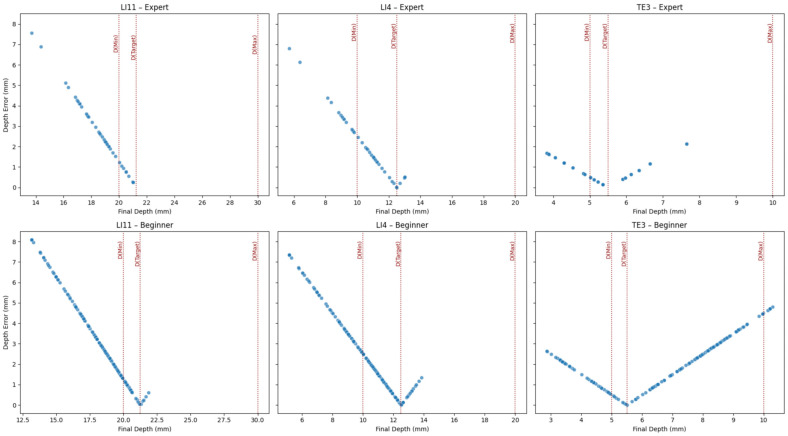
Scatter plots of final depth versus depth error for each acupoint and user group, with haptics enabled. Red dotted lines indicate expert-defined thresholds: minimum acceptable depth: D(Min), target depth: D(Target), and maximum safe depth: D(Max). Both expert and beginner users showed strong convergence toward the target region, guided by the haptic device.

To further assess the effectiveness of the wearable haptic device in aiding depth control during needle insertion, scatter plots of final depth versus depth error were generated for each acupoint and user group (see Figure 12). The haptic device was programmed to increase vibration intensity in proportion to the insertion depth and to emit a continuous buzz once the needle exceeded the maximum depth. The results show that both expert and beginner users were generally able to maintain their insertions within or close to the acceptable range, indicating effective utilization of haptic cues. For the LI11 and LI4 acupoints, depth estimates were well-distributed around the target region for both groups, although beginners exhibited slightly wider error margins. Notably, for the more challenging TE3 acupoint, which has a shallow depth range (target depth = 5.5 mm), a few beginner trials exceeded the maximum safe threshold. Still, the over-insertions remained minimal (within 1 mm). This suggests that the continuous vibration feedback effectively signaled excessive insertion to the users. However, the lack of distinct vibration cues at the *D_min_* and *D_target_* thresholds may explain the broader spread of depth errors, particularly among beginners who lacked clear tactile markers to identify when the ideal insertion depth was reached. In interpreting Figure 10 and Figure 12, insertions within the ±2 mm tolerance band were considered accurate, whereas those outside this range but within the minimum–maximum boundaries were regarded as safe but suboptimal. Overall, the results demonstrate that the vibrotactile guidance system helped users estimate needle depth accurately and safely, with minimal deviation from the optimal range.

**Figure 13:**
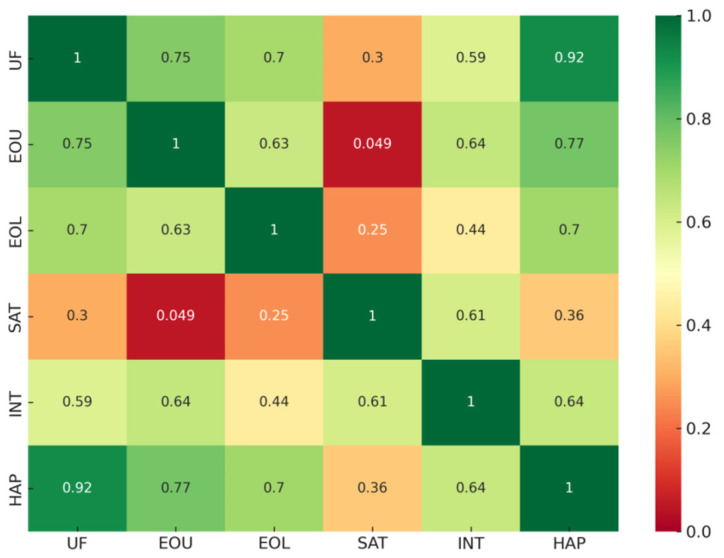
Correlogram of factor-level Spearman correlations (ρ) for the six usability dimensions of the MR acupuncture training system.

Following completion of both training modes, participants rated the system using the 15-item usability questionnaire. The descriptive statistics for each usability factor are summarized in Table 7, and inter-factor relationships are visualized in the correlogram (see Figure 13). Overall, ratings were high across all dimensions, with mean scores above 4.4 on the 5-point scale, indicating consistently positive user evaluations of the MR training experience.

Spearman correlation analysis revealed strong positive associations among most factors (ρ > 0.6), suggesting that perceptions of usefulness, ease of use, learning efficiency, and interaction realism were mutually reinforcing. The strongest relationship was observed between Usefulness and Haptic Feedback (ρ = 0.92), indicating that the haptic wrist-ring device was central to participants’ sense of the system’s practical training value. Similarly, correlations between Ease of Use and Haptic Feedback (ρ = 0.77) and between Ease of Use and Interaction Naturalism (ρ = 0.64) suggest that intuitive device control and realistic manipulation substantially contributed to user satisfaction. Satisfaction itself exhibited moderate associations with Interaction Naturalism (ρ = 0.61), but weaker links with other factors, implying that overall satisfaction may depend on experiential quality beyond operational simplicity.

Open-ended responses further reinforced these findings. Participants described the haptic cues as “realistic,” “informative,” and “helpful for judging depth,” though several noted mild discomfort from the wristband or HMD during extended use. Collectively, these results suggest that multisensory realism, particularly through tactile feedback, played a pivotal role in shaping the perceived effectiveness and acceptance of the training system.

These findings demonstrate that the MR acupuncture needling trainer is feasible, usable, and capable of producing measurable learning and procedural stability within short, repeated sessions. The results provide evidence for both the construct validity of the scoring model and the added value of haptic feedback for depth control. At the same time, user-reported measures confirm the system’s acceptability as a training platform.

## 4. Discussion

This study introduced and evaluated an MR acupuncture training platform incorporating a full-scale MetaHuman avatar, an expert-informed scoring algorithm, and a depth-responsive wearable haptic feedback device. The system demonstrated significant usability and early training effects among MR-familiar participants, offering a novel contribution to simulation-based acupuncture education. These findings highlight the simulator’s potential to improve procedural realism and skill acquisition by combining spatially anchored MR visualization with real-time haptic feedback and quantitative assessment.

This outcome aligns with prior XR-based medical training studies that have emphasized the importance of quantitative feedback and procedural accuracy improvement [4,5,6,7,8]. However, unlike earlier XR or AR acupuncture prototypes that lacked haptic or performance assessment capabilities [2,27,28,29], the present system integrates both quantitative scoring and tactile guidance. The observed learning progression and usability scores support the findings from Sung et al. [4] and Jongbloed et al. [7], who reported that immersive feedback loops enhance skill retention and confidence. Moreover, by employing expert-defined acupoint tolerances, this study adapts task-specific evaluation frameworks previously validated for surgery and anesthesia simulators [13,14,15,16,17] to the acupuncture domain. Thus, the results consolidate earlier XR evidence while addressing the literature-identified gaps in haptic realism and objective assessment for acupuncture training. These alignments with prior research further support the system’s potential as a validated training tool within broader XR-based medical education frameworks.

A key achievement of this work lies in the integration of expert-derived acupoint modeling with tolerance-based performance scoring. By using clinically informed depth, angle, and tip location thresholds, the system ensures that training reflects realistic procedural expectations. The inclusion of tolerance bands was instrumental in mitigating the inherent precision limitations of the HoloLens 2 while maintaining clinically meaningful evaluation fidelity. Furthermore, continuous expert involvement in the development of acupoint modeling, marker placement, and scoring logic helped preserve clinical validity while striking a balance between instructional rigor and technical feasibility.

Although the present system does not yet employ a formal adaptive learning algorithm, it promotes progressive skill refinement through iterative, feedback-driven practice. The combination of visual and haptic feedback allows users to self-correct and improve across sessions, functioning as an implicit adaptive mechanism. The modular design of the MR–haptic framework also enables future integration of algorithmic adaptation, such as difficulty scaling or personalized feedback based on performance trends.

The wearable haptic device played a pivotal role in enhancing depth awareness. Its real-time vibration pattern effectively guided users in regulating insertion depth, with continuous buzzing at over-insertion serving as a corrective cue. Despite differences between vibration-based feedback and the actual tactile sensations of skin penetration, participants reported that the cues were informative and natural, supporting intuitive depth regulation. Notably, the lightweight, wireless wrist-based design preserved freehand mobility and ergonomic comfort, avoiding the encumbrance typical of many haptic systems.

Additionally, using a highly realistic MetaHuman avatar improved psychological realism. Participants indicated that human-like visual fidelity increased engagement and could reduce anxiety about practicing on real patients, aligning with evidence that anthropomorphic virtual patients enhance emotional readiness and immersion in medical simulation. Collectively, these elements advance the pedagogical goals of immersive, quantitative, and multisensory training for acupuncture.

The usability study also revealed technical constraints associated with high-precision holographic simulation. The slender holographic needle exhibited visual instability and lag during rapid hand movements, primarily due to tracking latency and rendering delays of HoloLens 2. These limitations affected fine-motor alignment but were mitigated through the scoring logic, which evaluates the needle’s final position upon release, mirroring real-world assessment practices where final placement matters most.

Another factor influencing interaction quality was the participants’ familiarity with MR. Early pilot testing with MR-unfamiliar users yielded frequent interaction errors, including difficulty grasping the virtual needle and selecting UI elements. Consequently, the study intentionally used MR-experienced participants to focus on system feasibility rather than basic interface learning. However, this highlights the need for tutorial or onboarding modules to support novice users in future iterations.

Among measured parameters, angular deviation displayed greater variability than depth, particularly among beginners. This may stem from the lack of physical resistance in holographic simulations, unlike real tissue, which stabilizes the insertion angle. Addressing this issue may involve implementing virtual constraints or visual angle-maintenance guides to enhance angular consistency. Despite these technical challenges, the integration of tolerance-based evaluation successfully compensated for hardware imprecision, ensuring reliable scoring and maintaining educational validity within the operational limits of current MR hardware.

While the findings affirm the system’s feasibility and short-term training benefits, several study-specific limitations warrant discussion. The present evaluation involved MR-familiar participants rather than medical trainees, meaning that usability and interaction reliability were assessed in a technically competent but non-clinical population. Consequently, the educational transferability of the system to real training environments remains to be validated. Furthermore, the study focused on only three acupoints, which limits its generalizability across the full acupoint spectrum and anatomical diversity. The user interface and interaction design also require refinement to accommodate participants with varying levels of familiarity with head-mounted displays, as interaction complexity may pose barriers for first-time MR users. Additionally, the short-term study duration precludes conclusions about long-term skill retention or the transfer of learned motor patterns to real-world practice. Finally, the modest sample size of ten participants, including two experts, was not sufficient for inferential statistical testing or subgroup analysis. Nevertheless, this scale is typical for early-phase feasibility studies of mixed-reality training systems, which primarily aim to establish technical reliability and construct validity rather than educational generalizability.

Future work should extend validation to clinical cohorts of acupuncture trainees to evaluate educational transfer and curricular integration. Expanding the acupoint library, improving angular guidance features, and enhancing haptic fidelity to simulate tissue resistance gradients are critical next steps. Broader testing with MR-unfamiliar novices will ensure accessibility for diverse learner populations. Additionally, algorithmic personalization and adaptive feedback mechanisms may further optimize the learning curve. Once validated among medical students and integrated into curricula, the system could provide a standardized, scalable, and multisensory training platform for developing procedural skills in acupuncture.

Beyond acupuncture training, the haptic-MR framework developed here shares conceptual mechanisms with human–machine interaction systems, especially those applying impedance or admittance learning in contact tasks [33,34]. Thus, this wearable haptic-enabled MR method could be extended to domains such as tele-manipulation, robot-assisted rehabilitation, and human–robot contact learning. Future research could explore cross-domain applications of this lightweight haptic design in human–machine interaction and rehabilitation robotics, extending its impact beyond medical simulation.

## 5. Conclusions

This study presented an MR acupuncture training system that integrates a high-fidelity MetaHuman avatar, expert-informed acupoint modeling, a multi-parameter scoring algorithm, and a wearable haptic feedback device. The system demonstrated usability, procedural stability, and learning progression within a short training duration among MR-familiar participants. The incorporation of tolerance-based evaluation and vibration-guided depth feedback effectively mitigated hardware limitations of HoloLens 2, while the MetaHuman model enhanced the psychological realism of the training environment. The findings confirmed that the expert-weighted scoring framework reliably differentiated user proficiency and that repeated trials supported measurable improvement across depth, angle, and time metrics. However, challenges such as interaction complexity, angular instability, and limited haptic realism underscore areas for future enhancement. Broader validation with medical learners, expansion to diverse acupoints, and refinement of feedback modalities are necessary to confirm educational efficacy. Overall, this system represents a promising direction for immersive, quantitative, and feedback-driven acupuncture training within modern medical education frameworks.

## Figures and Tables

**Table 1:** Expert-derived marker dimensions and clinical parameters.

Acupoint	Min Depth (mm)	Ideal Depth (mm)	Max Depth (mm)	Depth Tolerance (mm)	Ideal Angle	Angle Tolerance	Target Diameter (mm)
TE3 (Zhongzhu)	5	5.5	10	±2	45–90	±15	3
TE5 (Waiguan)	10	12.5	20	±2	90	±15	3
LI4 (Hegu)	10	12.5	20	±2	90	±15	3
LI10 (Shousanli)	10	15	30	±2	90	±15	3
LI11 (Quchi)	20	21.25	30	±2	90	±15	3
SP10 (Xuehai)	20	21.25	30	±2	90	±15	3
ST34 (Liangqiu)	10	15	20	±2	90	±15	3
GB33 (Xiyangguan)	10	10	20	±2	90	±15	3
EX_LE2 (Heding)	10	15	20	±2	90	±15	3
EX_LE4 (Neixiyan)	10	15	20	±2	45	±15	3
ST35 (Dubi)	10	15	20	±2	45	±15	3
LR8 (Ququan)	20	21.25	30	±2	90	±15	3
SP9 (Yinlingquan)	20	21.25	30	±2	90	±15	3
GB34 (Yanglingquan)	20	21.25	30	±2	90	±15	3
ST36 (Zusanli)	20	21.25	30	±2	45–90	±15	3
GB39 (Xuanzhong)	10	11	20	±2	90	±15	3
LR3 (Taichong)	5	6	12	±2	45–90	±15	3
GB41 (Zulinqi)	5	6	12	±2	45–90	±15	3

**Table 2:** Participant demographics.

Participant ID	Group	Age Range	Gender	MR Familiarity	Clinical Background
B1–B8	Beginner	22–32	M/F	MR-familiar	No practical needling; only theoretical knowledge (books/videos)
E1–E2	Expert	26–36	M/F	MR-familiar	Medical students with prior acupuncture or anatomy-based needle experience

**Table 3:** Experiment design overview for all participants.

Session	Acupoints Tested	Trials per Acupoint	Total Trials per Session	Notes
Practice	Free exploration	–	–	30–60 min familiarization
Session 1	LI4, LI11, TE3	10	30	All trials with haptics ON
Session 2	LI4, LI11, TE3	10	30	Conducted after 24 h; haptics ON
Post-study Questionnaire	–	–	–	USE-based questionnaire, Likert + open-ended items

**Table 4:** Overall Mean (± SD) performance scores comparing beginners and experts.

Group	Depth Score (Mean ± SD)	Angle Score (Mean ± SD)	Final Score (Mean ± SD)
Beginners (n = 8)	89.09 ± 15.58	70.86 ± 34.65	56.26 ± 23.37
Experts (n = 2)	94.34 ± 10.94	75.49 ± 28.45	69.25 ± 21.64

**Table 5:** Early vs. late trial performance among beginners at each acupoint.

Acupoint	Metric	Early Mean ± SD (Trials 1–5)	Late Mean ± SD (Trials 16–20)
LI11	Depth (mm)	16.24 ± 1.88	19.74 ± 1.23
	Angle (°)	27.83 ± 16.62	15.34 ± 12.66
	Duration (s)	38.77 ± 14.68	13.28 ± 5.53
LI4	Depth (mm)	8.30 ± 1.90	11.72 ± 1.29
	Angle (°)	27.79 ± 15.73	14.84 ± 10.03
	Duration (s)	42.90 ± 18.17	12.05 ± 4.67
TE3	Depth (mm)	8.25 ± 1.77	5.63 ± 1.63
	Angle (°)	28.07 ± 14.20	15.91 ± 12.01
	Duration (s)	46.64 ± 19.47	13.03 ± 5.06

**Table 6:** Reduction in error rates and preparation time across training progression.

Metric	Early Mean (Trials 1–5)	Late Mean (Trials 16–20)
Needle Drops (count)	2.54	0.62
Wrong Attempts (count)	3.44	1.08
Preparation Time (s)	10.47	3.11

**Table 7:** Descriptive statistics for each usability factor.

Factor	Number of Questions	Mean ± SD	McDonald’s ω	Interpretation
UF	3	4.47 ± 0.47	0.86	High perceived performance support and learning value
EOU	3	4.45 ± 0.48	0.84	System operation and control were intuitive.
EOL	2	4.55 ± 0.50	0.81	Rapid familiarization and confidence in use
SAT	3	4.38 ± 0.59	0.79	General satisfaction and recommendation intention
INT	2	4.35 ± 0.49	0.82	Movements felt realistic and intuitive.
HAP	2	4.55 ± 0.52	0.88	Feedback felt informative and comfortable.
Overall Scale	15	4.46 ± 0.51	0.93	Excellent overall internal consistency

## Data Availability

Data collected for this study are presented in the manuscript.
